# Thoracic textilomas: CT findings*

**DOI:** 10.1590/S1806-37132014000500010

**Published:** 2014

**Authors:** Dianne Melo Machado, Gláucia Zanetti, Cesar Augusto Araujo, Luiz Felipe Nobre, Gustavo de Souza Portes Meirelles, Jorge Luiz Pereira e Silva, Marcos Duarte Guimarães, Dante Luiz Escuissato, Arthur Soares Souza, Bruno Hochhegger, Edson Marchiori

**Affiliations:** Department of Radiology, Antonio Pedro University Hospital, Fluminense Federal University, Niterói, Brazil; Graduate Program in Radiology, Federal University of Rio de Janeiro, Rio de Janeiro, Brazil; Department of Internal Medicine and Diagnostic Support, Federal University of Bahia, Salvador, Brazil; Federal University of Santa Catarina, Florianópolis, Brazil; Thoracic Radiology Team, Fleury Group, São Paulo, Brazil; Department of Internal Medicine and Diagnostic Support, Federal University of Bahia School of Medicine, Salvador, Brazil; Thoracic Imaging Section, Department of Imaging, AC Camargo Cancer Center and Heliópolis Hospital, São Paulo, Brazil; Department of Clinical Medicine, Federal University of Paraná, Curitiba, Brazil; São José do Rio Preto School of Medicine, São José do Rio Preto, Brazil; Federal University of Health Sciences of Porto Alegre, Porto Alegre, Brazil; Fluminense Federal University, Niterói, Brazil

**Keywords:** Foreign-body reaction, Tomography, spiral computed, Thoracic surgery

## Abstract

**OBJECTIVE:**

The aim of this study was to analyze chest CT scans of patients with thoracic textiloma.

**METHODS:**

This was a retrospective study of 16 patients (11 men and 5 women) with surgically confirmed thoracic textiloma. The chest CT scans of those patients were evaluated by two independent observers, and discordant results were resolved by consensus.

**RESULTS:**

The majority (62.5%) of the textilomas were caused by previous heart surgery. The most common symptoms were chest pain (in 68.75%) and cough (in 56.25%). In all cases, the main tomographic finding was a mass with regular contours and borders that were well-defined or partially defined. Half of the textilomas occurred in the right hemithorax and half occurred in the left. The majority (56.25%) were located in the lower third of the lung. The diameter of the mass was ≤ 10 cm in 10 cases (62.5%) and > 10 cm in the remaining 6 cases (37.5%). Most (81.25%) of the textilomas were heterogeneous in density, with signs of calcification, gas, radiopaque marker, or sponge-like material. Peripheral expansion of the mass was observed in 12 (92.3%) of the 13 patients in whom a contrast agent was used. Intraoperatively, pleural involvement was observed in 14 cases (87.5%) and pericardial involvement was observed in 2 (12.5%).

**CONCLUSIONS:**

It is important to recognize the main tomographic aspects of thoracic textilomas in order to include this possibility in the differential diagnosis of chest pain and cough in patients with a history of heart or thoracic surgery, thus promoting the early identification and treatment of this postoperative complication.

## Introduction

Textiloma (also known as gossypiboma) is a term used to describe the presence of a mass within a patient's body consisting of a cotton matrix, which usually corresponds to retained surgical gauze or sponge, surrounded by foreign body reaction.^(^ 1 ^-^ 3 ^)^ A textiloma is the most common retained surgical foreign body.^(^ 4 ^)^ It can occur in any part of the body and after any type of surgery, but most cases reported in the literature are linked to abdominal surgery.^(^ 5 ^)^ Few cases are related to thoracic surgery.^(^ 6 ^,^ 7 ^)^ Because it is a relatively uncommon condition, with serious medical-legal consequences and nonspecific clinical manifestations that can, however, be accompanied by severe complications (such as hemoptysis, fistulas, and abscesses), which are sometimes fatal,^(^ 8 ^-^ 10 ^)^ it is necessary that clinicians, surgeons, and radiologists always consider this diagnostic possibility and its radiological presentations so that early diagnosis and correct definitive treatment can be established in a timely manner.

In general, performing an X-ray is the first step, since X-ray is the cheapest and most readily available method and, in the presence of a visible radiopaque marker, it can be conclusive. Transthoracic or transesophageal ultrasound can be important in the assessment of a textiloma and its relationship with adjacent structures. Usually, CT is the most effective method to establish the diagnosis and to analyze possible associated complications.^(^ 11 ^)^ In cases of a textiloma in the pericardial cavity, magnetic resonance imaging allows a more appropriate and reliable assessment of the relationship between the mass under study and the heart wall, facilitating surgical planning.^(^ 12 ^-^ 14 ^)^

Since this is a benign condition, it is important that the correct diagnosis be established in the preoperative phase, because it allows surgical planning to be conducted more carefully, unlike what occurs in cases of acute lesions. It also allows surgeons to be more aware of possible associated complications that should be resolved during the same operation. In addition, because it behaves as a mass and mimics a neoplasm, patient anxiety and stress caused by an incorrect or inconclusive diagnosis should be taken into consideration. Although some cases of textiloma are clinically silent and are only discovered on routine radiological examination, surgical treatment is indicated in almost all cases,^(^ 15 ^)^ given that most patients have a frequent history of cough, chest pain, and hemoptysis. These complications generally resolve completely after resection of the lesion.

The objective of the present study was to investigate the main morphological characteristics and the most common CT findings of textilomas, as well as to analyze some epidemiological aspects of textilomas, such as clinical manifestations, gender and age distribution, and causal procedure.

## Methods

This was a retrospective, descriptive observational study of CT scans of 16 patients with a confirmed diagnosis of textiloma. The CT scans were randomly obtained from radiologists and thoracic surgeons at various medical institutions in six Brazilian states, between January of 2005 and October of 2013. In addition, we collected clinical and epidemiological data about those patients, including age, gender, signs and symptoms, causal procedure, and surgical location of the lesion.

We included patients (n = 16) in whom the diagnosis of textiloma was confirmed by surgical excision of the mass, the analysis of which revealed fragments of surgical sponge. Two of the patients had previously undergone transthoracic biopsy, but in only one of them were small filaments, described as foreign bodies, identified by histopathology. The study was approved by the Research Ethics Committee of the Antonio Pedro University Hospital of the Fluminense Federal University. Because this was a retrospective study, using existing clinical data with no change in patient monitoring or treatment, written informed consent from patients was not required.

No standardization existed in the CT studies because multiple institutions were involved and multiple CT scanners were used. However, all scans were acquired with slice thicknesses ranging from 1 to 10 mm, at 5- to 10-min intervals, from the apices to the hemidiaphragms or until the entire mass was included, for masses extending to the abdomen, with the patient in the supine position, and at end-inhalation. The images were acquired and reconstructed in a 512 × 512 matrix and photographed, for assessment of the lung fields, at a window width of 1,200 to 2,000 HU and a level of −300 to −700 UH. For assessment of the mediastinum, the images were photographed at a window width of 350 to 500 HU and a center of 10 to 50 HU. The scans were evaluated by two independent radiologists. Discordant results were resolved by consensus.

The masses on each of the scans were analyzed for the following characteristics: contour; borders; diameter; homogeneity; content; contrast enhancement; location (right or left hemithorax; lower, middle, or upper third of the hemithorax); origin (parietal, mediastinal, or pulmonary); and presence or absence of associated findings (pleural thickening, atelectasis, pleural effusion, and consolidation). The criteria used to define the CT findings were those reported in a Brazilian consensus.^(^ 16 ^)^ A mass was defined as any expansive pulmonary, pleural, mediastinal, or chest wall lesion with soft tissue, adipose tissue, or bony tissue density, greater than 3 cm in diameter, with at least partially defined contours, outside the fissural area, regardless of the characteristics of its contours or the heterogeneity of its content. Consolidation was defined as increased lung parenchymal attenuation that precludes the visualization of the vessels and outer contours of the bronchial walls. Atelectasis was defined as decreased lung volume due to lower aeration of part or all of the lung.

## Results

### Clinical and epidemiological aspects

The clinical manifestations of the 16 patients included chest pain, in 11 patients (68.75%); cough, in 9 (56.25%); dyspnea, in 4 (25.00%); pain in the right shoulder, in 2 (12.50%); and low-grade fever, in 2 (12.50%). One patient (6.25%) was asymptomatic. The patient group consisted of 11 men (68.75%) and 5 women (31.25%). Patient ages ranged from 29 to 85 years, with a mean of 52 years and 2 months. Regarding the original surgery, 10 patients (62.50%) had undergone heart surgery, 3 (18.75%) had undergone lung surgery, and 3 (18.75%) had undergone other procedures (repair of a diaphragmatic laceration, in 2; and mediastinal tumor resection, in 1). The interval between the original surgery and the diagnosis of textiloma ranged from 1 to 120 months, with a mean of 30.6 months (2.5 years).

### CT aspects

All 16 patients underwent CT. Iodinated contrast material was used in 13 patients (81.25%). The contrast material was not used in 3 patients (18.75%), because they had a history of allergy to iodine.

**Figure 1:**
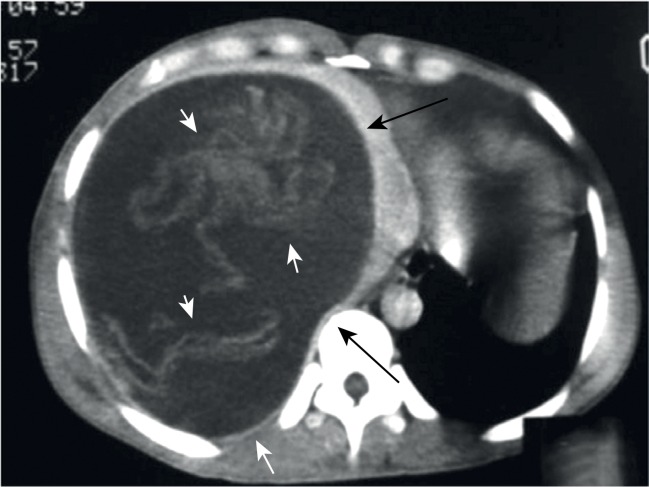
Intravenous contrast-enhanced chest CT scan with mediastinal window settings. Presence of a bulky cystic mass (arrows) with regular contours and well-defined borders, occupying and bulging the entire lower third of the right hemithorax and crossing the midline. Note peripheral contrast enhancement and typical folds (arrowheads) within the cystic mass, corresponding to the retained surgical sponge.

**Figure 2:**
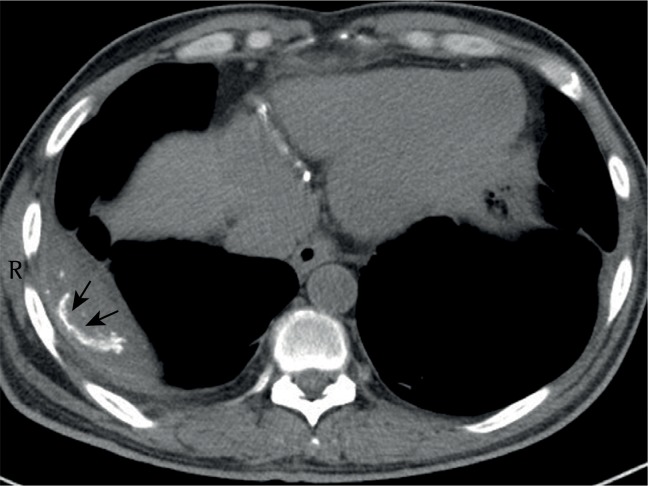
Non-contrast-enhanced chest CT scan with mediastinal window settings. Presence of an ovoid mass in the lower third of the right hemithorax, with soft tissue density, containing a dense linear image that corresponds to the retained surgical sponge marker (arrows). The lesion has regular contours and maintains, in most of its extent, close contact with the pleural surface.

**Figure 3:**
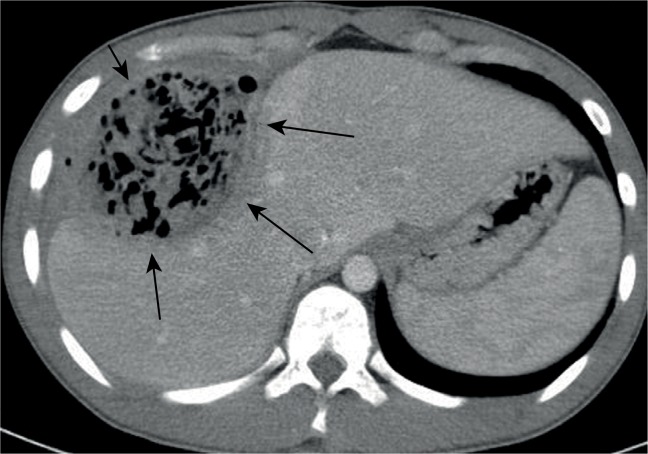
Axial intravenous contrast-enhanced chest CT scan with mediastinal window settings. Presence of a mass (arrows) with regular contours and welldefined borders at the base of the right hemithorax, compressing the liver and showing a typical spongiform pattern due to the presence of gas within it.

**Figure 4:**
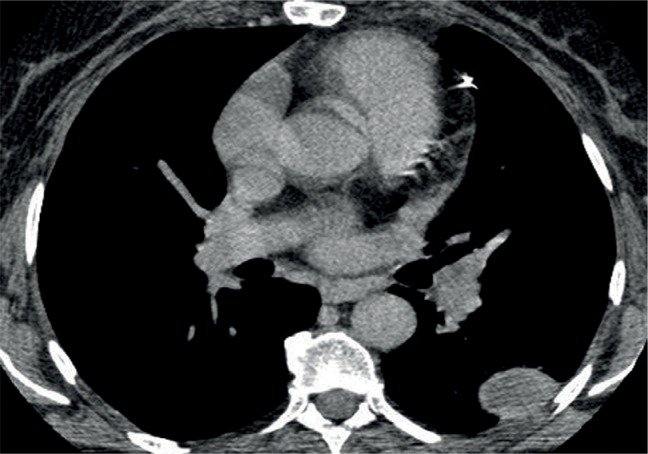
Axial intravenous contrast-enhanced chest CT scan with mediastinal window settings. Presence of a mass with regular contours, well-defined borders, and peripheral contrast enhancement, in close contact with the pleural surface and located posteriorly in the middle third of the left hemithorax.

All cases presented as a (round or ovoid) mass with regular contours and borders that were well-defined or partially defined. In 7 cases (43.76%), the mass measured no more than 5 cm in diameter; in 4 (25.00%), it measured 10-15 cm; in 3 (18.75%), it measured 5-10 cm; in 1 (6.25%), it measured 15-20 cm; and in 1 (6.25%), it measured 20-25 cm. Three (18.75%) of the 16 masses were homogeneous, and 13 (81.2%) were heterogeneous. It was possible to identify images consistent with sponge-like material in 6 (46.1%; Figure 1); radiopaque marker, in 3 (23.1%; Figure 2); gas permeating the foreign body, in 2 (15.4%; Figure 3); calcification, in 1 (7.7%); and other findings (high and low attenuation areas), in 6 (46.1%). Some masses had two or more simultaneous changes. We found peripheral enhancement of the mass (Figure 4) in 12 (92.3%) of the 13 patients in whom the contrast agent was used. No other type of enhancement was identified. A folded pattern was identified in 3 patients (18.7%), and a spongiform pattern was identified in 2 (12.5%).

Half (50.0%) of the textilomas occurred in the right hemithorax and half (50.0%) occurred in the left. The textilomas were located in the lower third of the chest in 9 patients (56.3%), in the middle third in 5 (31.2%), and in the upper third in 2 (12.5%). In addition to the mass, 13 patients (81.2%) had other CT findings, which included pleural thickening, in 6 patients (46.15%); atelectasis, in 5 (38.50%); pleural effusion, in 3 (23.10%); and parenchymal consolidation, in 1 (7.70%). No patients were identified with fistula.

### Surgical and pathological aspects

CT-guided transthoracic fine needle aspiration biopsy was performed in only 2 patients (12.5%). No neoplastic cells were identified in either of the biopsy samples. For only 1 of those 2 patients did the pathologist detect the presence of small filaments on the slide, described as foreign bodies. The 16 patients underwent surgery, and, in all of them, the presence of a foreign body containing cotton fibers was confirmed.

Sponge fragments, fibrous wall, and giant cell foreign body reaction were observed in all masses. In addition, areas of necrosis and calcification were identified in some of them. Regarding the space (pleural, pericardial, or mediastinal space) within which the mass was found intraoperatively, there was a significant predominance of the pleural space, in 14 (87.5%) of the 16 patients, followed by the pericardial space, in only 2 (12.5%).

## Discussion

In the present study, which deals with thoracic textilomas exclusively, we found a predominance of males, which corresponded to 68.75% of the cases. Because the vast majority of published papers on thoracic textilomas consist of isolated case reports, few describe the incidence of textilomas by gender. According to one group of authors,^(^ 17 ^)^ referring to textilomas in general, there is a slight predominance in females (63%), which is related to the fact that pelvic surgery is more common in this group than in males. However, in the case of thoracic textilomas, we found no reports of prevalence by gender in the literature.

As to prevalence by age, in our sample, the age group affected was the 29- to 85-year age group. We found no reports of prevalence by age in the literature. This is probably due to the fact that, unlike many diseases, textilomas can affect any age group, being not directly related to age, but rather directly related to the surgical procedures undergone by patients, which can occur at any age. An analysis of literature case reports involving adult patients showed that age at diagnosis of gossypiboma ranged from 22 years^(^ 3 ^)^ to 87 years.^(^ 18 ^)^ In the vast majority of cases, age at diagnosis does not correspond to age at onset of the mass, because although the diagnosis in some cases is established in a young patient, the surgery that caused the textiloma often was performed many years earlier, whereas in an elderly patient, the original surgery often was performed recently. In fact, in the above two references, we found that, despite being much younger, the 22-year-old patient had undergone the original surgery at 3 years of age, i.e., 19 years earlier, to treat a penetrating wound to the left hemithorax, whereas the patient who was diagnosed at 87 years of age had undergone the original surgery (aortic valve replacement) only 7 days prior. In addition, on the basis of these two cases, we note that the age range is very similar to that found in our study, as well as to that reported in most studies.

The most common complaints in our sample were chest pain, cough, and dyspnea. According to the literature, the most common clinical manifestations are cough, expectoration, chest pain, hemoptysis, and dyspnea.^(^ 3 ^,^ 19 ^,^ 20 ^)^ Regarding the surgery that caused the textiloma, heart surgery predominated, having been performed in 10 patients (62.5%), followed by lung surgery, in 3 (18.75%), and by other procedures (repair of a diaphragmatic laceration and mediastinal tumor resection), also in 3 (18.75%). In almost all published studies focused specifically on thoracic textilomas that we consulted,^(^ 2 ^,^ 3 ^,^ 12 ^-^ 14 ^,^ 20 ^-^ 26 ^)^ heart surgery predominated as the original surgery, followed by lung surgery.

In the present study, the interval between the original surgery and the diagnosis of textiloma ranged from 1 to 120 months, with a mean of 30.6 months. Because the condition can occur after any surgical procedure and because some patients are asymptomatic, we deduce that this interval can vary greatly. Therefore, on the basis once again of published research focusing exclusively on thoracic textilomas, by means of a report-by-report analysis, we could confirm this variability. There are patients in whom the diagnosis was established shortly after the original surgery, as in the report by Whang et al.,^(^ 18 ^)^ in which the interval was 7 days. In other patients, the diagnosis was made long after the original surgery, as in the report by Madan et al.,^(^ 13 ^)^ in which the interval between the original surgery and the diagnosis was 46 years. Even considering these details, it is not always possible to determine exactly how long the surgical sponge has been in the patient's body. Some patients cannot accurately tell the date of the original surgery or how old they were at the time. Other patients underwent more than one surgical procedure, which makes it difficult to determine the exact time of the onset of the condition. One group of authors^(^ 3 ^)^ reported the case of a female patient who had undergone at least two thoracic procedures, making it impossible to determine which intervention had caused the textiloma.

CT scan analysis showed that, in all of our 16 cases, the mass had a similar shape, as well as regular contours and borders that were mostly well defined. In most of the papers consulted,^(^ 3 ^,^ 14 ^,^ 20 ^,^ 23 ^)^ textilomas were described as masses, and no other morphological aspects were identified. In addition, it is of note that the lesions are almost always regular and well defined.^(^ 3 ^,^ 7 ^,^ 20 ^,^ 25 ^)^

In most cases in our sample (87.5%), the mass measured 4 to 15 cm in diameter. As was the case with gender and age, we found no statistically significant values for mean diameter of thoracic textilomas in the literature. Analysis was made even more difficult by the fact that many authors^(^ 3 ^,^ 12 ^,^ 23 ^)^ have emphasized several radiological characteristics of the lesion (homogeneity, as well as presence of gas, calcifications, and markers) in their papers, but without mentioning its diameter. In the studies in which the diameter of the mass was reported,^(^ 2 ^,^ 7 ^,^ 13 ^,^ 20 ^-^ 22 ^,^ 24 ^)^ the vast majority of the masses measured 4 to 9 cm, although one was reported to measure 14 cm.^(^ 14 ^)^ These data are very similar to our findings.

Significant CT characteristics in our sample included heterogeneity, in 13 (81.2%) of the 16 cases, and peripheral enhancement, in 12 (92.3%) of the 13 cases in which intravenous contrast material was used. There is a consensus among authors, especially among those who have published papers highlighting CT findings of thoracic textilomas, that chief among the predominant characteristics of these lesions are heterogeneity and peripheral enhancement.^(^ 3 ^,^ 7 ^,^ 27 ^)^

The lesions that were heterogeneous in density showed findings consistent with sponge-like material, gas, calcification, or radiopaque marker. Of those aspects, the one most commonly found in the cases of the present study was sponge-like material, which was seen in 6 of the 16 masses. Gas bubbles were found within 2 lesions only. Some authors have shown that, despite being typical, the finding of air bubbles trapped in the fibers of the surgical sponge can be absent. ^(^ 3 ^,^ 28 ^,^ 29 ^)^ A surgical sponge left in the pleural space, which corresponded to 87.5% of the cases in our sample, does not typically result in images of gas because of the reabsorption of air by the pleura,^(^ 7 ^,^ 12 ^,^ 13 ^)^ which usually occurs within the first 30 days after surgery.^(^ 30 ^)^ Therefore, bubbles may not be a prominent finding in retained intrathoracic surgical sponges, as is the case of those in the abdominal cavity. In the consulted literature, there were no specifications as to the frequency at which sponge-like material, gas, calcifications, or radiopaque markers are identified within textilomas.

A folded pattern was identified in 3 of the 16 cases in our sample, and a spongiform pattern was identified in 2. The air crescent sign, described on rare occasions,^(^ 21 ^)^ was not seen in any of the cases in our sample. There also have been no reports in the literature of the frequency of spongiform or folded patterns. 

It is important to note that some authors, in a retrospective analysis, found that certain characteristics of the mass changed over the years, while the diagnosis was not confirmed. This refers not only to characteristics such as homogeneity, given that an initially homogeneous textiloma can become heterogeneous after calcifications or gas develop within it, but also to others, since there may be changes in size and even location when it comes to pleural masses.^(^ 19 ^,^ 21 ^)^

Neither hemithorax was predominantly affected, but the lower third was involved in most cases (56.25%). No frequency rates for these data were available in the articles studied. Associated findings were present in 13 patients (81.2%). Pleural thickening, atelectasis, and pleural effusion were found in 6 (37.5%), 5 (31.2%), and 3 (18.8%) of the patients, respectively.

It was possible to establish that, on the basis of case reports in the literature, in the vast majority of cases, the diagnosis of thoracic textiloma is confirmed only after surgical resection.^(^ 3 ^,^ 19 ^,^ 21 ^)^ This finding is in agreement with ours, since in only 1 of the 16 patients studied was the diagnosis confirmed by transthoracic biopsy before resection.

The most common site of occurrence of textilomas was the pleural space (in 14 of the 16 cases; 87.5%). The pericardial space was involved in only 2 cases (12.5%), with the lesion being located posteriorly. These data corroborate those reported in the literature. Some authors have shown that the thoracic site where retained surgical sponges are most commonly found is the pleural space, followed by the pericardial space.^(^ 2 ^,^ 7 ^,^ 12 ^)^ Even in studies in which the initiating surgical procedure was heart surgery, the pleural space was the most commonly affected site.^(^ 20 ^)^ When the pericardial space is involved, the mass tends to be located posteriorly,^(^ 3 ^,^ 23 ^)^ which also occurred in our sample.

In conclusion, in this study, the cases of thoracic textiloma presented as masses, mostly heterogeneous, with peripheral contrast enhancement, and neither hemithorax was predominantly affected. The most common clinical manifestations were chest pain and cough. In the majority of the cases, the initiating surgical procedure was heart surgery, and most textilomas were found intraoperatively in the pleural space.
