# Supplementary material for: Thoracic textilomas: CT findings
Source: J Bras Pneumol. 2014 Sep-Oct;40(5):535–42. doi: 10.1590/S1806-37132014000500010 (PMC4263335; doi:10.1590/S1806-37132014000500010)
Supplement: Translation

# Translation: Textilomas intratorácicos: achados tomográficos*

**Authors:** Dianne Melo Machado, Gláucia Zanetti, Cesar Augusto Araujo, Luiz Felipe Nobre, Gustavo de Souza Portes Meirelles, Jorge Luiz Pereira e Silva, Marcos Duarte Guimarães, Dante Luiz Escuissato, Arthur Soares Souza, Bruno Hochhegger, Edson Marchiori

**Affiliations:** Serviço de Radiologia, Hospital Universitário Antonio Pedro, Universidade Federal Fluminense, Niterói (RJ) Brasil; Programa de Pós-Graduação em Radiologia, Universidade Federal do Rio de Janeiro, Rio de Janeiro (RJ); e Professora de Clínica Médica. Faculdade de Medicina de Petrópolis, Petrópolis (RJ) Brasil; Departamento de Medicina Interna e Apoio Diagnóstico, Universidade Federal da Bahia, Salvador (BA) Brasil; Universidade Federal de Santa Catarina, Florianópolis (SC) Brasil; Equipe de Radiologia Torácica, Grupo Fleury, São Paulo (SP); e Professor Orientador. Programa de Pós-Graduação, Departamento de Diagnóstico por Imagem, Universidade Federal de São Paulo, São Paulo (SP) Brasil; Departamento de Medicina Interna e Apoio Diagnóstico, Faculdade de Medicina da Bahia, Universidade Federal da Bahia, Salvador (BA) Brasil; Setor de Imagem Torácica, Departamento de Imagem, AC Camargo Cancer Center e Hospital Heliópolis, São Paulo (SP) Brasil; Departamento de Clínica Médica, Universidade Federal do Paraná, Curitiba (PR) Brasil; Faculdade de Medicina de São José do Rio Preto, São José do Rio Preto (SP) Brasil; Universidade Federal de Ciências da Saúde de Porto Alegre, Porto Alegre (RS) Brasil; Universidade Federal Fluminense, Niterói (RJ); e Professor Associado. Universidade Federal do Rio de Janeiro, Rio de Janeiro (RJ) Brasil

**Keywords:** Reação a corpo estranho, Tomografia computadorizada espiral, Cirurgia torácica

## Abstract

**OBJETIVO:**

Analisar retrospectivamente os aspectos encontrados em TCs de tórax com textiloma torácico.

**MÉTODOS:**

Estudo retrospectivo de 16 pacientes (11 homens e 5 mulheres) com diagnóstico de textiloma torácico confirmado cirurgicamente. As TCs de tórax foram avaliadas, de modo independente, por dois observadores, e os casos discordantes foram resolvidos por consenso.

**RESULTADOS:**

Na maioria dos casos (62,5%), o fator causal foi a cirurgia cardíaca prévia. Os sintomas mais frequentes foram dor torácica (em 68,75%) e tosse (em 56,25%). Em todos os casos, o principal achado tomográfico foi de massa com contornos regulares e limites bem definidos ou parcialmente definidos. A localização dos textilomas ocorreu na mesma proporção no hemitórax direito e esquerdo, mas foi mais comum no terço inferior (em 56,25%). O tamanho das massas foi ≤ 10 cm e > 10 cm, respectivamente, em 10 (62,5%) e em 6 pacientes (37,5%). A maioria dos textilomas apresentou densidade heterogênea (81,25%), observando-se no seu interior calcificações, gás, marcador radiopaco ou material da compressa. A impregnação periférica da lesão foi observada em 12 (92,3%) dos 13 pacientes que receberam o meio de contraste. A cirurgia demonstrou acometimento do espaço pleural e pericárdico, respectivamente, em 14 (87,5%) e em 2 pacientes (12,5%).

**CONCLUSÕES:**

É importante reconhecer os principais aspectos tomográficos dos textilomas intratorácicos a fim de incluir essa possibilidade no diagnóstico diferencial em pacientes com dor torácica e tosse e história de cirurgia cardíaca ou torácica, contribuindo assim para o tratamento precoce dessa complicação cirúrgica.

## Introdução

Textiloma (também chamado de gossipiboma ou tecidoma) é um termo usado para descrever a presença de uma massa dentro do corpo composta de uma matriz de algodão, que usualmente corresponde a uma gaze ou compressa cirúrgica retida, circundada por uma reação de corpo estranho.^(^ 1 ^-^ 3 ^)^ Textiloma é o corpo estranho cirúrgico retido mais comum.^(^ 4 ^)^ Pode ocorrer em qualquer parte do corpo e após qualquer tipo de cirurgia, mas a maioria dos casos relatados na literatura está ligada à cirurgia abdominal.^(^ 5 ^)^ Poucos casos são relacionados à cirurgia torácica.^(^ 6 ^,^ 7 ^)^ Por tratar-se de uma afecção relativamente incomum, com sérias implicações médico-legais e manifestações clínicas inespecíficas que, entretanto, podem-se acompanhar de graves complicações (como hemoptise, fístulas e abscessos), algumas vezes fatais,^(^ 8 ^-^ 10 ^)^ faz-se necessário que o clínico, o cirurgião e o radiologista tenham sempre em mente essa hipótese diagnóstica e suas formas de apresentação radiológica para que um diagnóstico precoce e um tratamento correto e definitivo sejam feitos em tempo hábil.

A radiografia, por ser o método mais barato e mais facilmente acessível, em geral é o primeiro exame realizado e, na presença de um marcador radiopaco visível, poderá ser conclusiva. A ultrassonografia transtorácica e/ou transesofágica pode ser importante na avaliação do textiloma e de sua relação com as estruturas adjacentes. A TC costuma ser o método mais eficaz na realização do diagnóstico e na análise de possíveis complicações associadas.^(^ 11 ^)^ No caso de um textiloma na cavidade pericárdica, a ressonância magnética permite uma avaliação mais adequada e confiável da relação da massa em estudo com a parede cardíaca, facilitando o planejamento cirúrgico.^(^ 12 ^-^ 14 ^)^

É importante que o diagnóstico correto seja feito ainda na fase pré-operatória, pois, por tratar-se de uma condição benigna, permite que o planejamento cirúrgico seja elaborado com mais cuidado, diferentemente do que ocorre quando as intervenções são realizadas em lesões agudas. Permite também que o cirurgião tenha uma noção mais adequada de possíveis complicações associadas que devam ser resolvidas no mesmo ato cirúrgico. Além disso, por comportar-se como uma massa e simular neoplasia, a ansiedade e o estresse do paciente ante um diagnóstico incorreto ou inconclusivo devem ser levados em consideração. Embora alguns casos de textiloma sejam clinicamente silenciosos e somente descobertos em exames radiológicos de rotina, o tratamento cirúrgico está indicado na quase totalidade dos casos,^(^ 15 ^)^ já que os pacientes, em sua maioria, apresentam história frequente de tosse, dor torácica e hemoptise. Tais complicações são, em geral, completamente sanadas após a ressecção da lesão.

O objetivo do presente estudo foi avaliar as principais características morfológicas dos textilomas e os seus achados tomográficos mais frequentes, além de analisar alguns de seus aspectos epidemiológicos, como manifestações clínicas, distribuição por sexo e idade e cirurgia causal.

## Métodos

Foi realizado um estudo observacional descritivo retrospectivo das TCs de 16 pacientes com diagnóstico confirmado de textiloma. Esses exames foram reunidos aleatoriamente com radiologistas e cirurgiões torácicos de várias instituições médicas em seis estados brasileiros, no período compreendido entre janeiro de 2005 e outubro de 2013. Foi realizado também um levantamento dos dados clínicos e epidemiológicos desses pacientes, que incluiu o registro da idade, gênero, sinais e sintomas, cirurgia causal e localização cirúrgica das lesões.

Foram incluídos no estudo pacientes com o diagnóstico de textiloma confirmado através de excisão cirúrgica (n = 16) da massa, cuja análise revelou fragmentos de compressa. Dois dos pacientes foram antes submetidos à biópsia transtorácica, mas somente em um deles foram identificados na histopatologia pequenos filamentos descritos como corpos estranhos. O estudo foi aprovado pelo Comitê de Ética em Pesquisa do Hospital Universitário Antonio Pedro da Universidade Federal Fluminense. Como o estudo foi retrospectivo, usando os dados clínicos existentes sem nenhuma mudança no acompanhamento e/ou tratamento dos pacientes, não foi necessário o consentimento informado por parte dos mesmos.

Não houve padronização nas rotinas dos estudos tomográficos devido às múltiplas instituições e tomógrafos envolvidos. Contudo, todos os exames foram realizados com cortes variando de 1 a 10 mm de espessura, com intervalos de 5 a 10 mm, dos ápices às cúpulas diafragmáticas ou até incluir toda a massa, no caso das que se estendiam ao abdome, com o paciente em decúbito dorsal e em fase final de inspiração. As imagens foram obtidas e reconstruídas em matriz de 512 × 512 e fotografadas para a avaliação dos campos pulmonares com aberturas de janela variando de 1.200 a 2.000 UH e nível variando entre −300 e −700 UH. Para o estudo do mediastino, a variação das janelas foi de 350 a 500 UH, e a de centro, entre 10 e 50 UH. Os exames foram avaliados, de forma independente, por dois radiologistas. Nos casos discordantes o resultado final foi obtido por consenso.

Em cada um dos exames foram analisadas as características das massas quanto ao contorno, limites, tamanho, homogeneidade, conteúdo, impregnação pelo meio de contraste, localização em relação ao hemitórax e o respectivo terço comprometido, origem (parietal, mediastinal ou pulmonar), e presença ou não de achados associados (espessamento pleural, atelectasia, derrame pleural e consolidação). Os critérios utilizados para definir os achados tomográficos foram aqueles relatados em um consenso brasileiro. ^(^ 16 ^)^ A massa foi definida como qualquer lesão expansiva pulmonar, pleural, mediastinal ou da parede torácica, com densidade de partes moles, de gordura ou óssea, maior que 3 cm, com contornos pelo menos parcialmente definidos, fora de topografia cissural, independentemente das características de seus contornos ou da heterogeneidade de seu conteúdo. A consolidação foi definida como um aumento da atenuação do parênquima pulmonar que impede a visualização dos vasos e dos contornos externos das paredes brônquicas. A atelectasia foi definida como uma redução volumétrica do pulmão decorrente de uma menor aeração de uma parte ou de todo o pulmão.

## Resultados

### Aspectos clínicos e epidemiológicos 

As manifestações clínicas dos 16 pacientes incluíram dor torácica, em 11 pacientes (68,75%); tosse, em 9 (56,25%); dispneia, em 4 (25,00%); dor no ombro direito, em 2 (12,50%); e febre baixa, em 2 (12,50%). Um paciente (6,25%) era assintomático. Em relação ao sexo, o grupo de pacientes foi composto por 11 homens (68,75%) e 5 mulheres (31,25%). A idade dos pacientes variou de 29 a 85 anos, com uma média de 52 anos e 2 meses. Quanto à cirurgia de origem, 10 pacientes (62,50%) haviam sido submetidos à cirurgia cardíaca, 3 (18,75%) à cirurgia pulmonar e 3 (18,75%) a outros procedimentos (correção de laceração diafragmática, em 2, e ressecção de tumor mediastinal, em 1). O intervalo de tempo entre a cirurgia de origem e o diagnóstico do textiloma variou de 1 a 120 meses, com uma média de 30,6 meses (2,5 anos).

### Aspectos na TC

Todos os 16 pacientes foram submetidos à TC. O meio de contraste iodado foi administrado em 13 pacientes (81,25%). Por apresentarem história de alergia ao iodo, o meio de contraste não foi administrado em 3 pacientes (18,75%).

Todos os casos apresentaram-se como massa (redonda ou ovalada), de contornos regulares, com limites bem definidos ou parcialmente definidos. Em 7 casos (43,76%) a massa media, no máximo, 5 cm de diâmetro; em 4 (25,00%), essa media entre 10-15 cm; em 3 (18,75%), entre 5-10 cm; em 1 (6,25%), entre 15-20 cm; e, em 1 (6,25%), entre 20-25 cm. Três (18,75%) das 16 massas eram homogêneas, e 13 (81,2%) eram heterogêneas. Foi possível identificar imagens compatíveis com material com aspecto de algodão de compressa em 6 (46,1%; Figura 1); marcador radiopaco, em 3 (23,1%; Figura 2); gás de permeio ao corpo estranho, em 2 (15,4%; Figura 3); calcificações, em 1 (7,7%); e outros achados (áreas com alta e baixa atenuação), em 6 (46,1%). Algumas massas tinham duas ou mais alterações simultâneas. Observamos impregnação periférica da lesão (Figura 4) em 12 (92,3%) dos 13 pacientes nos quais foi administrado o meio de contraste. Nenhum outro tipo de impregnação foi identificado. O padrão pregueado foi identificado em 3 pacientes (18,7%), e o espongiforme, em 2 (12,5%).

**Figura 1:** TC de tórax com janela para o mediastino, com contraste intravenoso. Presença de volumosa massa cística (setas) de contorno regular e limites bem definidos, ocupando e abaulando todo o terço inferior do hemitórax direito e cruzando a linha média. É possível notar o realce periférico pela substância de contraste e o típico pregueado (cabeças de seta) em seu interior, correspondendo à compressa retida.

**Figura 2:** TC de tórax com janela para o mediastino, sem contraste intravenoso. Presença de massa ovalada no terço inferior do hemitórax direito, com densidade de partes moles, contendo imagem linear densa que corresponde ao marcador (setas) da compressa retida. A lesão tem contorno regular e mantém, em sua maior extensão, íntimo contato com a superfície pleural.

**Figura 3:** TC de tórax, corte axial, com janela para o mediastino e contraste intravenoso. Presença de massa (setas) com contorno regular e limites bem definidos na base do hemitórax direito, exercendo compressão sobre o fígado e apresentando o típico padrão espongiforme pela presença de gás no seu interior.

**Figura 4:** TC de tórax, corte axial, com janela para o mediastino e contraste intravenoso. Presença de massa de contorno regular, com limites bem definidos, impregnação periférica pelo meio de contraste, em íntimo contato com a superfície pleural, localizada posteriormente no terço médio do hemitórax esquerdo.

Quanto ao hemitórax comprometido, metade das lesões (50,0%) localizava-se à direita, e a outra metade, à esquerda (50,0%). As lesões localizavam-se no terço inferior do tórax em 9 pacientes (56,3%); no terço médio, em 5 (31,2%); e no terço superior, em 2 (12,5%). Em 13 pacientes (81,2%) havia, além da massa, outros achados à TC, que incluíram espessamento pleural, em 6 pacientes (46,15%); atelectasia, em 5 (38,50%); derrame pleural, em 3 (23,10%); e consolidação do parênquima, em 1 (7,70%). Em nenhum caso foi identificado trajeto fistuloso.

### Aspectos cirúrgicos e anatomopatológicos 

Foi realizada biópsia transtorácica com agulha fina guiada por TC em apenas 2 pacientes (12,5%). Nenhuma delas identificou células neoplásicas. Somente em 1 desses 2 pacientes o patologista notou a presença de pequenos filamentos na lâmina, descritos como corpos estranhos. Os 16 pacientes foram submetidos à cirurgia, e em todos foi confirmada a presença de corpo estranho com material de algodão.

Foram observados fragmentos de compressa, parede fibrosa e reação de corpo estranho com células gigantes em todas as massas. Em algumas foram identificadas, ainda, áreas de necrose e calcificação. Quanto ao espaço (pleural, pericárdico ou mediastinal) em que foi encontrada a massa no ato cirúrgico, notou-se um importante predomínio do espaço pleural, observado em 14 (87,5%) dos 16 pacientes, estando as 2 (12,5%) restantes no espaço pericárdico.

## Discussão

No presente estudo, que trata exclusivamente dos textilomas torácicos, notou-se um predomínio do sexo masculino, que correspondeu a 68,75% dos casos. Como a grande maioria dos artigos publicados sobre textilomas torácicos restringe-se a relatos de casos isolados, poucos descrevem a incidência dos textilomas segundo o sexo. De acordo com um grupo de autores,^(^ 17 ^)^ referindo-se aos textilomas em geral, há um leve predomínio no sexo feminino (63%), relacionado ao fato de esse grupo ser submetido a cirurgias pélvicas com mais frequência que o masculino. Entretanto, no caso dos textilomas torácicos, não foram encontrados relatos de prevalência segundo o sexo na literatura consultada.

Quanto à prevalência por idade, na nossa casuística a faixa etária acometida variou entre 29 e 85 anos. Nenhum relato de prevalência por idade foi encontrado na literatura. Isso se deve, provavelmente, ao fato de que a ocorrência dos textilomas, diferentemente de muitas doenças, não apresenta uma predileção por faixa etária, não estando diretamente relacionada à idade, mas sim aos procedimentos cirúrgicos a que são submetidos os pacientes, o que pode ocorrer em qualquer faixa etária. A análise dos relatos de casos em pacientes adultos encontrados na literatura demonstra que a idade de diagnóstico do gossipiboma variou entre 22 anos^(^ 3 ^)^ e 87 anos.^(^ 18 ^)^ Na imensa maioria das vezes, a idade ao diagnóstico não corresponde à idade da ocorrência do textiloma, pois embora o diagnóstico, em alguns casos, seja feito num paciente jovem, muitas vezes a cirurgia que originou o textiloma foi realizada muitos anos antes, enquanto que num paciente idoso, muitas vezes, a cirurgia original aconteceu recentemente. Desse modo, tomando os dois casos supracitados como exemplos, observamos nos relatos que na paciente de 22 anos, embora bem mais jovem, a cirurgia original foi realizada aos 3 anos de idade, ou seja, 19 anos antes, para o tratamento de uma ferida penetrante no hemitórax esquerdo, enquanto, no segundo exemplo (paciente diagnosticado aos 87 anos), a cirurgia original, para substituição de válvula aórtica, havia sido realizada há apenas 7 dias. Usando ainda esses dois casos como referência, nota-se que a amplitude dessa faixa etária é muito semelhante à nossa, assim como à maioria dos casos relatados na literatura.

As queixas mais comuns dos nossos pacientes foram dor torácica, tosse e dispneia. De acordo com os dados da literatura, as manifestações clínicas mais comuns são tosse, expectoração, dor torácica, hemoptise e dispneia.^(^ 3 ^,^ 19 ^,^ 20 ^)^ Quanto à cirurgia que levou à ocorrência do textiloma, houve um predomínio de cirurgias cardíacas, que foi a cirurgia realizada em 10 pacientes (62,5%); seguida pela pulmonar, em 3 (18,75%); e por outros procedimentos (correção de laceração diafragmática e ressecção de tumor mediastinal) também em 3 (18,75%). Em quase todos os trabalhos publicados e consultados especificamente sobre textilomas torácicos,^(^ 2 ^,^ 3 ^,^ 12 ^-^ 14 ^,^ 20 ^-^ 26 ^)^ a cirurgia original predominante também foi a cardíaca, seguida pela pulmonar.

No estudo atual, o intervalo de tempo entre a cirurgia original e o diagnóstico do textiloma variou de 1 a 120 meses, com uma média de 30,6 meses. Como a afecção pode surgir após qualquer procedimento cirúrgico e como alguns pacientes são assintomáticos, deduzimos que esse intervalo pode ser muito variável. Assim, tomando por base, mais uma vez, as publicações exclusivas sobre textilomas torácicos e analisando relato por relato, pudemos confirmar essa variabilidade. Há pacientes cujo diagnóstico foi feito pouco tempo depois da cirurgia original, como no relato de Whang et al.,^(^ 18 ^)^ cujo período foi de 7 dias. Em outros pacientes, o diagnóstico foi realizado muito tempo depois da cirurgia original, como no relato de Madan et al.,^(^ 13 ^)^ cujo intervalo entre a cirurgia original e o diagnóstico foi de 46 anos. Mesmo considerando esses detalhes, nem sempre é possível definir exatamente há quanto tempo a compressa estava no corpo do paciente. Alguns pacientes não sabem dizer com precisão a data ou a idade que tinham na época da cirurgia original. Outros pacientes foram submetidos a mais de um procedimento cirúrgico, dificultando a identificação do exato momento da ocorrência dessa afecção. Um grupo de autores^(^ 3 ^)^ relatou o caso de uma paciente submetida há pelo menos dois procedimentos torácicos, não sendo possível determinar qual intervenção levou à ocorrência do textiloma.

Ao estudo tomográfico, a forma de massa foi comum em todos os nossos 16 casos, assim como o contorno regular e limites, em sua maioria, bem definidos. Na maior parte dos artigos consultados,^(^ 3 ^,^ 14 ^,^ 20 ^,^ 23 ^)^ os autores descreveram o textiloma sob a forma de massa, não tendo sido identificado nenhum outro aspecto morfológico. Ressaltaram, ainda, que a lesão quase sempre é regular e bem definida.^(^ 3 ^,^ 7 ^,^ 20 ^,^ 25 ^)^

Na maior parte dos nossos casos (87,5%), a massa mediu de 4 a 15 cm. Assim como em relação ao gênero e à idade, não se encontrou na literatura nenhum valor estatisticamente válido quanto ao tamanho médio dos textilomas torácicos. Dificultou ainda mais essa análise o fato de que muitos autores^(^ 3 ^,^ 12 ^,^ 23 ^)^ deram ênfase, em seus relatos, a várias características radiológicas da lesão (homogeneidade, assim como presença de gás, de calcificações e de marcador), mas sem citar seu tamanho. Nos estudos nos quais foi descrito o tamanho da massa,^(^ 2 ^,^ 7 ^,^ 13 ^,^ 20 ^-^ 22 ^,^ 24 ^)^ a imensa maioria media de 4 a 9 cm, havendo entretanto um relato de massa que media 14 cm.^(^ 14 ^)^ Esses dados são muito similares aos que encontramos.

As características tomográficas marcantes nos nossos casos foram a heterogeneidade, presente em 13 (81,2%) das 16 massas, e a impregnação periférica, observada em 12 (92,3%) dos 13 pacientes nos quais se administrou o meio de contraste intravenoso. Há concordância entre os autores, especialmente entre aqueles que publicaram trabalhos destacando os achados tomográficos dos textilomas torácicos, que, entre as características predominantes dessas lesões, destacam-se sua heterogeneidade e sua impregnação periférica.^(^ 3 ^,^ 7 ^,^ 27 ^)^

No interior das lesões heterogêneas foram identificadas imagens que correspondiam ao material da compressa, gás, calcificações ou marcador radiopaco. Dentre esses aspectos, o mais observado nos casos do presente estudo foi o do material da compressa, presente em 6 das 16 massas. Bolhas de gás foram encontradas no interior de somente 2 lesões. Alguns autores demonstraram que, embora típica, a presença de bolhas de ar aprisionadas entre as fibras da compressa pode estar ausente.^(^ 3 ^,^ 28 ^,^ 29 ^)^ Uma compressa deixada no espaço pleural, o que correspondeu a 87,5% dos nossos casos, geralmente não mostra imagens de gás devido à reabsorção do ar pela pleura,^(^ 7 ^,^ 12 ^,^ 13 ^)^ o que costuma ocorrer nos primeiros 30 dias depois da cirurgia.^(^ 30 ^)^ Portanto, as bolhas podem não ser um achado proeminente nas compressas intratorácicas retidas, como ocorre com aquelas na cavidade abdominal. Na literatura consultada, não havia especificação da frequência com que se podem identificar o material da compressa, gás, calcificações ou marcador radiopaco no interior dos textilomas.

O padrão pregueado foi observado em 3 dos nossos 16 pacientes, e o espongiforme, em 2. O sinal do crescente aéreo, descrito em raríssimas ocasiões,^(^ 21 ^)^ não foi visto em nenhum dos nossos casos. Os padrões espongiforme e pregueado também não tiveram relatos de frequência na literatura.

É importante registrar que alguns autores, numa análise retrospectiva, observaram que certas características da massa mudaram ao longo dos anos, enquanto seu diagnóstico não era confirmado. Isso se refere não somente a características, tais como homogeneidade, já que um textiloma inicialmente homogêneo pode passar a ser heterogêneo após o surgimento, em seu interior, de calcificações ou de gás, mas também a outras, uma vez que pode haver mudanças de tamanho e até de localização no que se refere às massas pleurais.^(^ 19 ^,^ 21 ^)^

Não houve predileção da lesão por hemitórax, mas o terço inferior foi acometido na maioria dos casos (56,25%). Nenhum índice de frequência quanto a esses dados estava disponível nos artigos estudados. Achados associados estavam presentes em 13 pacientes (81,2%). Espessamento pleural, atelectasia e derrame pleural foram encontrados em 6 (37,5%), 5 (31,2%) e 3 (18,8%) dos pacientes, respectivamente.

Foi possível constatar, pelos relatos de casos da literatura, que a grande maioria dos textilomas torácicos tem seu diagnóstico confirmado somente após a ressecção cirúrgica.^(^ 3 ^,^ 19 ^,^ 21 ^)^ Esse dado foi comum aos nossos casos, uma vez que, dos 16 pacientes estudados, somente 1 teve o diagnóstico confirmado, através de biópsia transtorácica, antes da ressecção.

Quanto à localização mais comum da ocorrência dos textilomas, o espaço pleural foi comprometido na maior parte dos casos (14 dos 16 casos; 87,5%). O espaço pericárdico foi comprometido somente em 2 casos (12,5%) e, nesses, em localização posterior. Esses dados corroboram os descritos na literatura. Alguns autores demonstraram que o sítio torácico mais comum de se encontrar uma compressa cirúrgica retida é o espaço pleural, seguido pelo pericárdico. ^(^ 2 ^,^ 7 ^,^ 12 ^)^ Até mesmo em estudos cujas cirurgias de origem foram as cardíacas, o espaço pleural foi o mais afetado.^(^ 20 ^)^ Quando o espaço pericárdico é o comprometido, a localização da massa tende a ser posterior,^(^ 3 ^,^ 23 ^)^ o que também ocorreu em nossa casuística.

Em conclusão, os casos de textilomas torácicos no presente estudo apresentaram-se sob a forma de massas, em sua maioria heterogêneas, com impregnação periférica pelo meio de contraste e sem predomínio quanto ao hemitórax. As manifestações clínicas mais frequentes foram dor torácica e tosse. A cirurgia original que mais comumente levou à ocorrência dos textilomas foi a cardíaca, e a maior parte dos textilomas foi cirurgicamente encontrada no espaço pleural.
